# Supplementary material for: Decoupling Choice from Motor Response Reduces Choice-History Effects
Source: bioRxiv. 2026 Jul 8:2026.07.02.736214. Preprint. [Version 1] doi: 10.64898/2026.07.02.736214 (PMC13370911; doi:10.64898/2026.07.02.736214)
Supplement: Supplement 1 [file NIHPP2026.07.02.736214v1-supplement-1.pdf]

bioRxiv preprint doi: https://doi.org/10.64898/2026.07.02.736214; this version posted July 8, 2026. The copyright holder for this preprint (which was not certified by peer review) is the author/funder. This article is a US Government work. It is not subject to copyright under 17 USC 105 and is also made available for use under a CC0 license.

Supplementary Figures

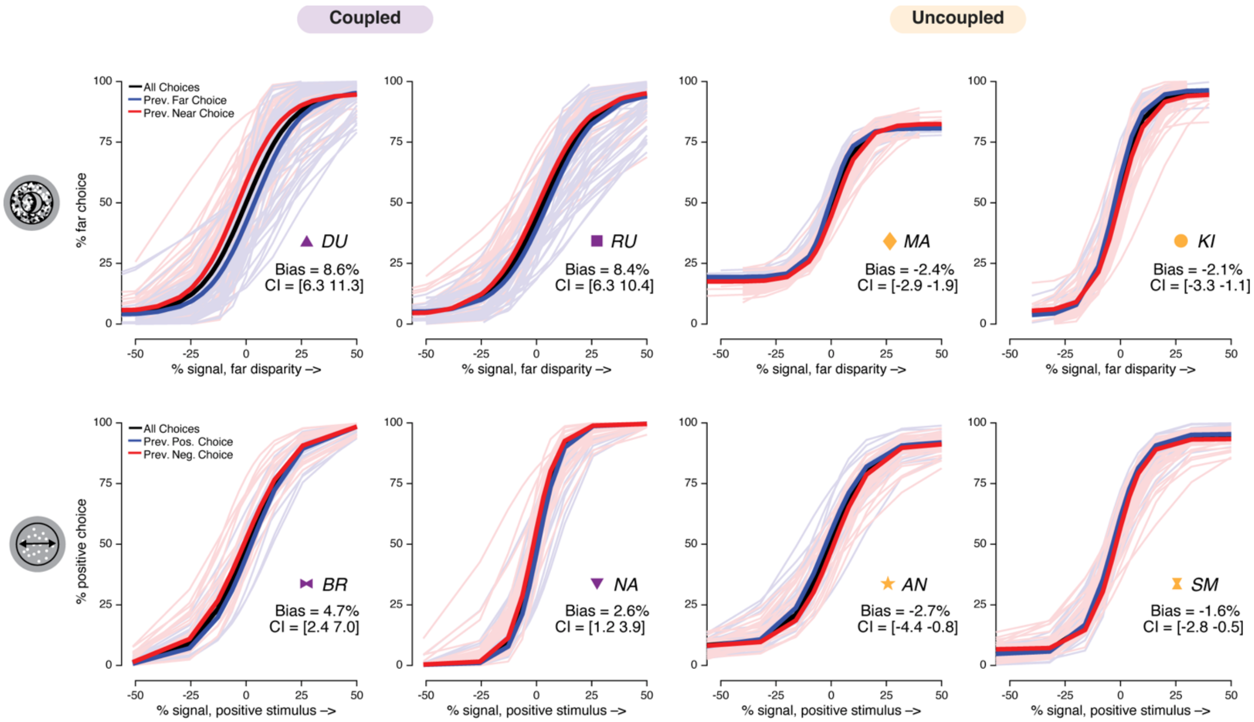

Supplementary Figure 1: Choice-History Conditioned Psychometric Curves for individual animals.

Here, we plotted choice-history conditioned psychometric curves for all four animal performing the disparity discrimination task (top row) and the motion discrimination task (bottom row). Each animal is denoted by a two-letter abbreviation and shape-icon. Transparent traces represent individual session choice-history conditioned fits. Thick trace represents the mean fit across sessions. Please see **Figure 1** and **Materials and Methods** for details on icon-animal mapping, model-fitting, sign-convention, and quantification of choice-history effects. “Bias” indicates mean choice-history effects across sessions and “CI” indicates the 95% bootstrap confidence interval for the mean bias.

bioRxiv preprint doi: https://doi.org/10.64898/2026.07.02.736214; this version posted July 8, 2026. The copyright holder for this preprint (which was not certified by peer review) is the author/funder. This article is a US Government work. It is not subject to copyright under 17 USC 105 and is also made available for use under a CC0 license.

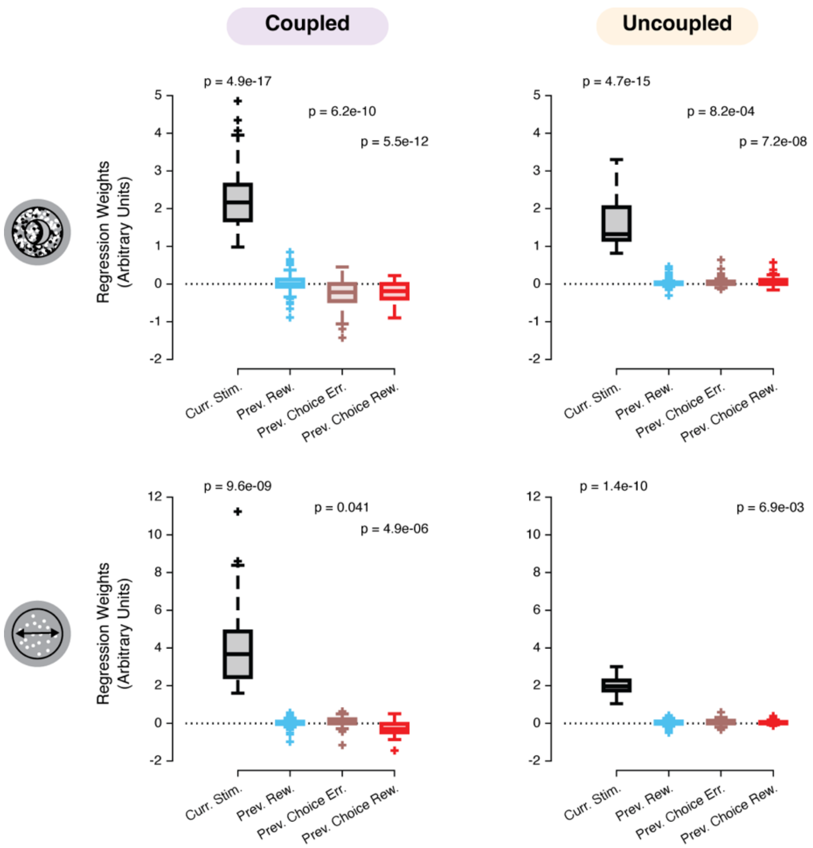

Supplementary Figure 2: Model weights describing choice history are larger in coupled animals

We fit a cross-validated generalized-linear model using regressors representing stimulus information and choice history. We obtained regression coefficients for all regressors within each session. Here, we plotted the regression coefficients for the stimulus and history parameters across sessions for each task variant (top row, disparity discrimination task; bottom row, motion discrimination task). History regressors, whose weights differ significantly from zero do not necessarily provide a significant boost in choice correlation (compare **Fig. 3**). History regressors that significantly increase choice correlation had negative regression coefficient weights. It indicates that the animals tend to alternate their choices. Boxplots show the distribution of model regression coefficients across sessions conditioned by task-variant. Box plot: whiskers, non-outlier maximum and minimum; crosses, outliers; horizontal line, median.

bioRxiv preprint doi: https://doi.org/10.64898/2026.07.02.736214; this version posted July 8, 2026. The copyright holder for this preprint (which was not certified by peer review) is the author/funder. This article is a US Government work. It is not subject to copyright under 17 USC 105 and is also made available for use under a CC0 license.

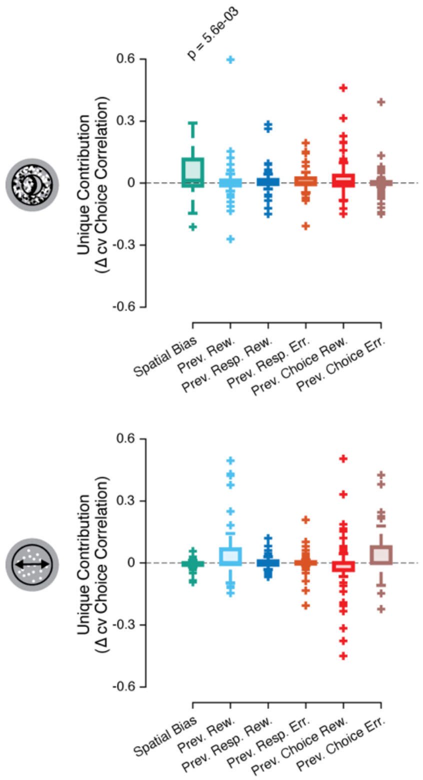

Supplementary Figure 3: Previous motor responses and spatial biases do not account for differences in history effects between task-variants

We quantify the unique contribution to choice prediction performance of each regressor in the expanded choice and response history model (see **Materials and Methods**). Box plot: whiskers, non-outlier maximum and minimum; crosses, outliers; horizontal line, median. Bonferroni-corrected p-values represent the significance of contribution to choice prediction performance by that particular regressor (Wilcoxon Signed-Rank test).
